# Quality improvement project to reduce length of stay for patients with urinary tract infections in an NHS hospital trust

**DOI:** 10.1136/bmjoq-2024-002998

**Published:** 2025-08-04

**Authors:** Molly Crawford

**Affiliations:** 1 Health Foundation, London, UK; 2 Frimley Health NHS Foundation Trust, Frimley, Surrey, UK

**Keywords:** Ambulatory care, Continuous quality improvement, Control charts/Run charts, Cost-effectiveness, Length of Stay

## Abstract

The bed day reduction improvement project for patients with urinary tract infections was commissioned at Frimley Health NHS Foundation Trust as inpatient length of stay (LOS) has been increasing over time, with noticeable variance between conditions and treatment pathways.

A multidisciplinary group was formed with staff from infection control, urology and medicine. A3 thinking (a quality improvement method) was used to define the problem, analyse the data, complete root cause analysis and test change.

The project aimed to impact the whole hospital system; however, using quality improvement methodology, the area with the biggest potential impact was focused on which was the emergency department. This is because positive changes made at the front end cause better outcomes throughout the pathway. Change ideas included reducing urine sample errors by improving labelling, increasing the number sent off by making the sample collection process easier for staff, increasing the use of Same Day Emergency Care Unit (SDEC) to avoid unnecessary admissions by raising awareness of the pathway with doctors and designing a pathway direct from triage to SDEC.

A link was demonstrated, through audit, between sample errors/not sent and prolonged LOS, confirming the opportunity of reducing sample errors. White-topped urine sample errors reduced by 50% following the process change. The work done to reduce errors has led to an approximate 10 days per month bed day saving, improving patient experience, care and staff morale. There was no significant increase in urine samples sent, the urology SDEC use increased marginally and the triage pathway was implemented. The project was unable to link the individual changes to a reduction in the outcome measure of bed days.

**WHAT IS ALREADY KNOWN ON THIS TOPIC**

- Prolonged length of stay (LOS) is well recognised to lead to negative patient outcomes such as deconditioning or increased likelihood of hospital-acquired infections.

**WHAT THIS STUDY ADDS**

- This project demonstrates that using cost-free simple process changes can change behaviour, reduce errors and lead to sustainable change.

**HOW THIS STUDY MIGHT AFFECT RESEARCH, PRACTICE OR POLICY**

- The process changes outlined in this project are simple, affordable and effective and therefore provide a ready system for implementation elsewhere in healthcare. Also, the audited link between urine sample errors and prolonged LOS gives evidence to focus on increasing successful sample rates or conducting further research on the link.

## Problem

### Hospital organisation background information

Frimley Health NHS Foundation Trust provides NHS hospital services for around 900,000 people across Berkshire, Hampshire, Surrey and south Buckinghamshire. The Trust has three main hospitals - Frimley Park Hospital (FPH) in Frimley near Camberley, Heatherwood in Ascot and Wexham Park Hospital (WPH) near Slough. 

Frimley Health’s inpatient length of stay (LOS) has been increasing over time, with noticeable variance between conditions and treatment pathways. LOS for patients with urinary tract infections (UTIs) increased 48% from 6.87 days (2021–2022) to 11.20 days (2022–2023). An average of 120 UTI patients is admitted with an average of 1350 bed days per month.

The potential impact of increased LOS on patients is deconditioning, risk of hospital-acquired infections and emotional stress of being away from home. Also, each additional bed day costs the organisation approximately £260^1^ (bed prices can change based on acuity of patient and the nursing ratios required) and adds pressure to bed availability and hospital flow.

#### Project vision

Patients with UTIs are only staying in hospital as long as clinically needed without unnecessary delays to discharge and patients are treated in a locational alternative to the emergency department (ED) where appropriate.

#### Project goal

Implement change initiatives (referred to as countermeasures) within 8 weeks of the project group’s first meeting and achieve a reduction in bed days for age 16–65 by 10%.

## Background

Between 2018 and 2023, 1.8 million hospital admissions were attributed to UTIs and therefore put a significant strain on hospital resources.^1^ An increased LOS can negatively impact patients. One study has shown that a LOS in hospital over 10 days can lead to 10 years of muscle ageing for patients who are aged over 80 years.^2^ The issue of prolonged LOS exists across the NHS (National Health Service), with several national targets being in place. For example, the 2023/2024 operational planning guidance had a target of reducing adult general and acute bed occupancy to 92% or below.^3^ A past initiative was NHS England’s priority to reduce patients staying over 21 days by 40% by March 2020 through the ‘where best next’ campaign, a UK initiative to improve patient discharge planning. Getting It Right First Time,^4^ a UK national programme to reduce unwarranted variation in care, particularly post-surgery, focused on reducing unwanted variation in LOS by standardising pathways and supporting hospital organisations to conform to best practice.

LOS projects have been conducted at other hospitals and an article set in a hospital in Singapore on a 21-bedded surgical ward aimed to reduce contamination of urine samples. It achieved a 40% reduction by conducting specific nurse education sessions and using an audit tool to monitor the change before and afterwards. It showed that education combined with using best practice processes improved the quality of sample collection and demonstrated practical applications to achieve success.^5^

While another study retrospectively reviewed urine cultures and antibiotic susceptibility results of patients over 17 years of age in an outpatient clinic between 2014 and 2018 covering 9556 urine samples. Resistance to ciprofloxacin, one of the most commonly used antibiotics for UTI, increased from 17 to 43% over the period and as well as for other common antibiotics. This further demonstrates how essential it is for patients’ urine to be tested before prescribing antibiotics to ensure a timely recovery in hospital.^6^

Regarding LOS, an article based on the English health system analysed 32 370 discharge episodes to determine whether there was a correlation between increased LOS and a higher readmission rate. The conclusion was that readmissions within 28 days were lower than expected (The study found that readmissions within 28 days were lower than expected (O:E ratio <1) for patients with shorter LOS (<4.3 days) but higher than expected for those with longer LOS across all ages.^7^ This backs up the hypothesis of the project that reducing the LOS of UTI patients is unlikely to produce increased readmissions.

## Measurement

The organisation’s digital analytics team supplied monthly LOS data for UTI patients throughout the project which included each patient’s LOS and the total number of admissions for the month, and therefore total bed days. Several other metrics were included, such as the age of the patient and the site they were discharged from. The data were inputted by clinicians into the electronic patient record and were subsequently coded for reporting purposes.

Upon team discussion, it was identified that the number of bed days would be a more effective measure as one of the potential countermeasures was admission avoidance; therefore, it would not impact overall LOS. The doctor in training conducted an initial patient audit, identifying a higher potential impact on the 16–65 age group who were more likely to be discharged home without complex social care issues. Therefore, the outcome measure was agreed to be monthly bed days for patients aged 16–65. A baseline was taken from 23 July to 24 August inclusive with an average of 139 total bed days per month for the age group with the condition.

## Design

The project team was led by an ED middle-grade doctor in training and consisted of a multidisciplinary team of doctors, urology specialist nurses, medical nurses, infection control nurses and a continuous improvement coach. Additional stakeholders were engaged based on the three countermeasures that were identified with additional detail below.

**Figure 1:**
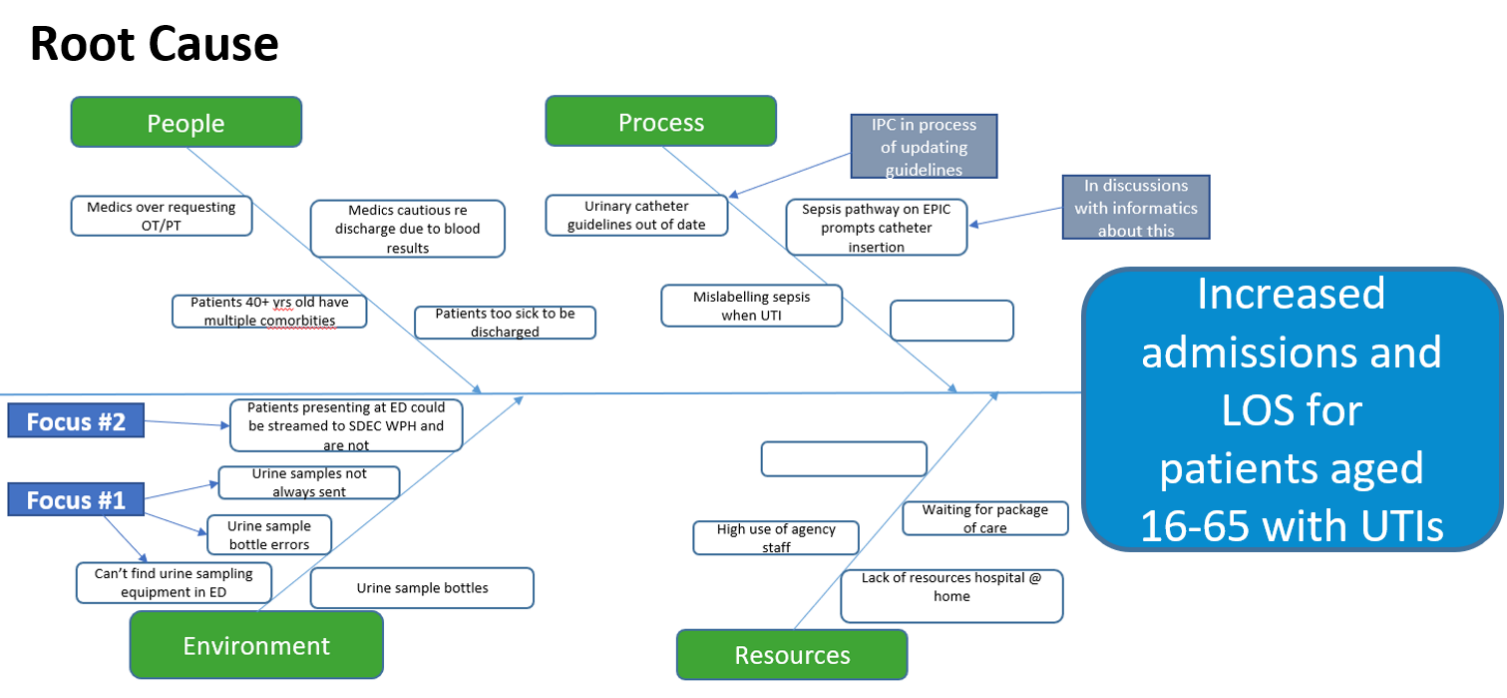
Root cause analysis of the problem of increased admissions and length of stay (LOS) for patients aged 16–65 with urinary tract infections (UTIs).

Following a root cause analysis of the factors contributing to a prolonged LOS, it was decided that the group focuses energies on factors that are in the group’s control and fit with the project timescale of 8 weeks to implementing countermeasures (figure 1).

### Countermeasures identified

**Figure 2:**
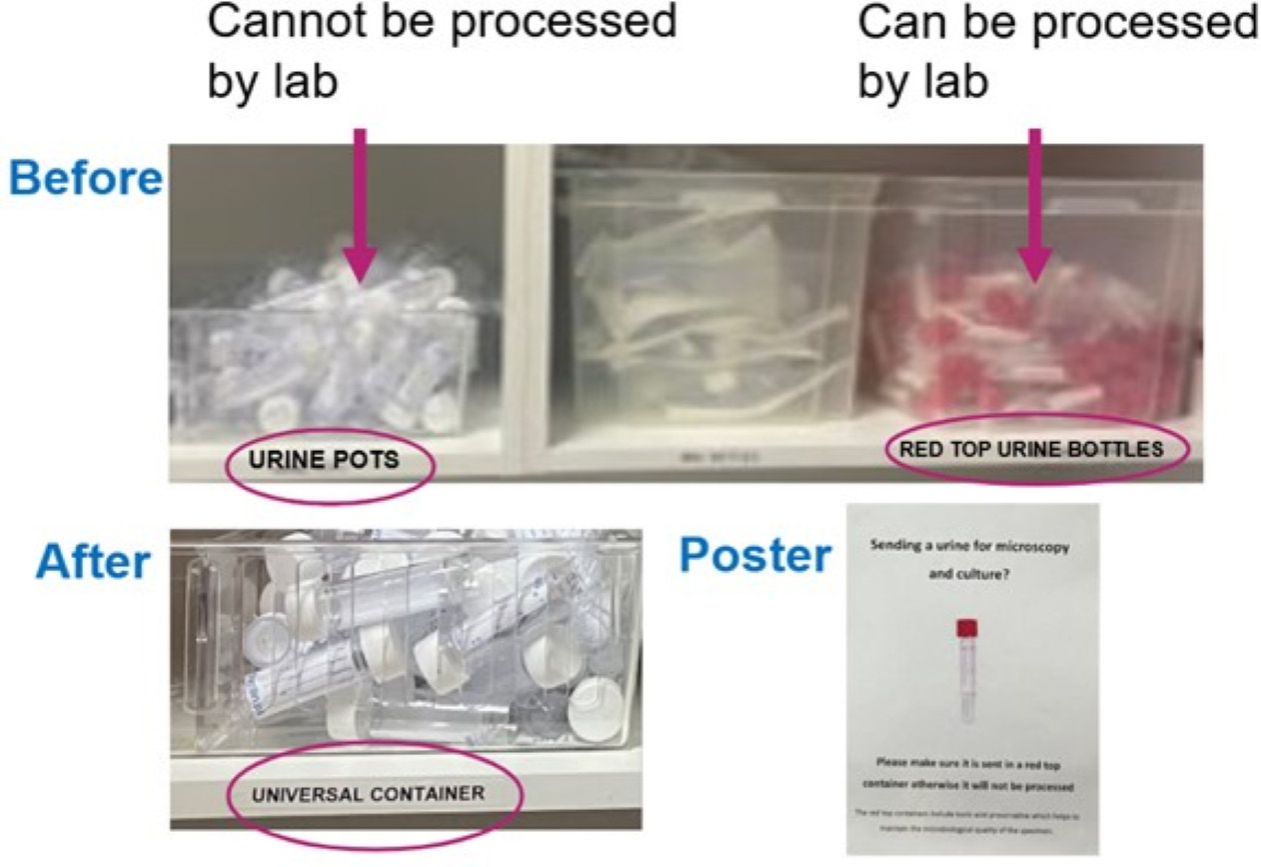
Urine labelling and poster change.

Countermeasure 1 was to reduce urine sample errors in the hospital. A urology middle-grade doctor in training identified anecdotally that sample errors were high at the Frimley Park Hospital site's ED. Laboratory data confirmed samples were being sent off in white-topped bottles rather than the red ones (figure 2). These cannot be processed as they do not contain the boric acid needed to preserve the urine for microscopy and culture for accurate analysis. Based on an audit of patients whose sample was sent but an error occurred so they did not have results, it was shown that over 50% of patients admitted they had a significantly prolonged LOS (approximately 6.8 days added), including one likely avoidable readmission due to prescription of infection-resistant antibiotics which did not treat the infection. Based on this link, the impact of addressing this root cause would be likely to contribute to the goal of reducing bed days.

Plan-Do-Study-Act (PDSA) 1: the labels were confusing for staff preparing urine samples with both bottle tubs stating, ‘urine bottles’. In order to reduce urine sample errors in ED, labelling in the waste disposal area was made clearer. Working with ED staff, the label for the white-topped bottles was changed from ‘urine bottles’ to ‘universal container’. The aim was to use a process change to change behaviour to reduce errors.

PDSA 2: to support education of staff, a poster was then put up in the area where the staff carried out the urine tests and prepared the samples. The aim was to increase awareness and therefore reduce errors. However, it is noted that posters have limited influence on behaviour change as observed in an experiment on university washrooms where putting up a poster caused hand hygiene compliance rates to increase by just 4% from 51% to 56%.^8^ Therefore, it was recognised that this intervention alone would not be sufficient as it was supplementary.

PDSA 3: nurses in charge and senior doctors reminded staff to use the new process through verbal reminders and through email. The aim was to increase awareness and therefore reduce errors.

PDSA 4: a process was put in place where if a data point was above the upper control limit, the project manager would alert the nursing staff who would investigate the cause and mitigate it going forward. The aim was to pick up issues and correct practice, reducing errors.

**Figure 3:**
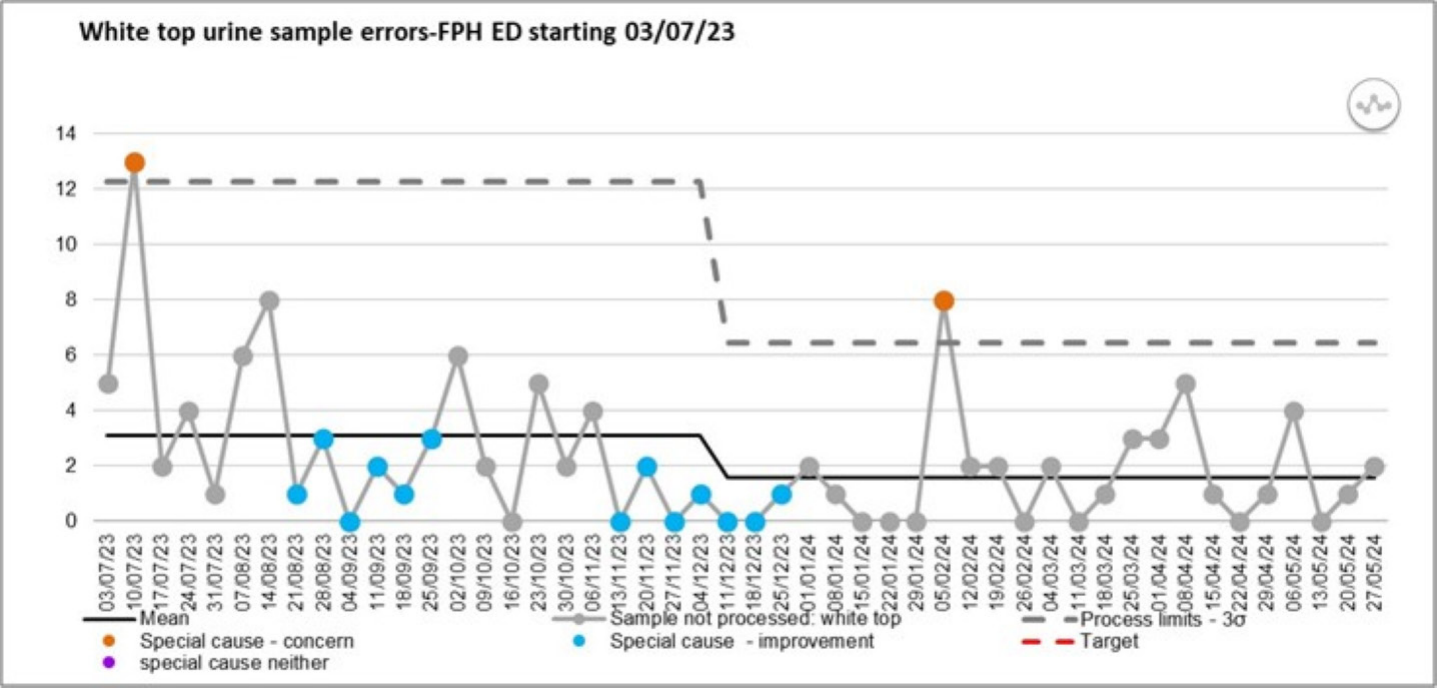
Statistical process control chart of urine sample errors due to being sent off in white tops which cannot be processed by the laboratory. Frimley Park Hospital ED, emergency department.

This was measured through monthly data provided by the pathology service, entered into a weekly statistical process control chart and monitored over the course of 25 weeks (figure 3).

Countermeasure 2 was related to the above but with a focus on increasing the urine samples sent for patients who had a suspected UTI. Following a patient audit, it was identified that at Wexham Park 65% (n=28) and Frimley Park 73% (n=33) of suitable patients had a sample sent against an expectation of 100%. The benefit of increasing samples sent would be the same as for reducing non-processed samples as with the previous countermeasure. The infection control nurse reviewed the current process with ED staff and concluded that a new and more streamlined process could improve the number of samples sent.

PDSA 1: the new process, including a change of sample pot, was agreed with the practice development senior nurses and two ED nurses in charge. The aim was to simplify the process to increase samples sent by staff and make it safer, reducing errors.

PDSA 2: nurses in charge and senior doctors then educated staff during floor-walks and clinical handovers to use the new process and how the change will benefit staff and patients. Additionally, they used email reminders to reinforce the education. The aim was that the percentage of samples sent would increase.

PDSA 3: following some queries on the new process, the infection control nurse lead made various visits to ED to ensure the equipment was in place and the staff were satisfied with the process. The aim was that the percentage of samples sent would increase and minimise errors due to unfamiliarity of the new process.

Initial feedback was collected verbally from the medics and via an online survey tool from the nursing staff. An audit was conducted before and after the PDSA cycles had been implemented.

Finally, countermeasure 3 related to increasing use of the Urology Same Day Emergency Care Unit (SDEC). An audit identified several patients admitted under urology who were eligible to be seen in the SDEC; therefore, the admission could have been avoided. A simple flow chart was completed, and qualitative data were received from a urology specialist nurse. The process was reported as working well at Frimley Park; therefore, the countermeasure was Wexham Park focused. It aimed to raise awareness at clinical handovers and teaching days and introduce a urology SDEC process of referring from ED to form departmental policy.

PDSA 1: raising awareness at ED clinical handovers of the criteria and benefit of using urology SDEC was carried out. Monthly data on the number of urology patients seen in Wexham Park SDEC showed a positive trend. The aim was to increase referrals to the Urology SDEC.

PDSA 2: following awareness raising, it was recognised that staff did not have a specific criteria to follow, making decision-making difficult and hindering the number of patients sent to SDEC, and so a new ED policy was written and ratified regarding Urology SDEC referrals so nurses can refer from triage and doctor in training can refer to SDEC. The policy was published in May 2024 and is being used by the triage nurse. In terms of education, a poster was placed in the triage area; governance meetings and clinical handovers were used to communicate the changes. Nurses also had a flow chart to follow during triage. The aim was to increase referrals to Urology SDEC and avoid admissions, reducing bed days.

Changing ED policy was a longer-term change which we do not have outcome data; however, the new pathway is operational, and the detail is on the nurse in charge desk in the ED for triage.

Balancing measures included staff satisfaction and workload burden and sample error rate due to other factors such as leakage and labelling which are explained further below.

#### Stakeholder engagement

Stakeholder engagement was a key element of the project, ensuring alignment and motivation across the team. Engagement strategies included monthly meetings, in-person visits and process mapping exercises to identify gaps and opportunities, fostering a shared understanding of the need for change. The first 5 min of each meeting was dedicated to quality improvement (QI) education, supplemented by individual QI training sessions. Team members were given ownership of PDSA cycles, which encouraged accountability and investment in the process. To further incentivise participation, certificates and formal recognition were awarded at the project’s conclusion. Senior leaders championed the initiative, reinforcing its importance and impact.

Challenges included the limited availability of resident doctors, which was mitigated by visiting them on their wards and providing administrative support. Resourcefulness was key, with engaged doctors from previous projects invited to contribute.

The engagement strategy proved effective, with all countermeasures implemented successfully. Many team members demonstrated self-motivation, and project outcomes were strong across most areas. While the PDSA cycles led by doctors had slightly less measurable impact due to limited data, their contributions were valuable in driving the project forward.

## Results

The first countermeasure saw average weekly errors reduce from 3.09 to 1.56 (50% decrease), including 7 weeks of 0 errors. The new average has been sustained for 25 weeks and we can say that the label created a positive step change in the process. Based on the strong connection between sample errors and increased LOS, the work done to reduce errors is forecasted to have led to an estimated 10 days per month bed day saving, improving patient experience, care and staff morale ^2^(based on assumptions from the sample error audit regarding prolongation of LOS). Overall, UTI LOS increased by 10%; however, the 16–65 bed days decreased by 10% over this period, due to the hard work and also increasing SDEC use and increasing urine samples sent. The urology specialty has always thought there to be a connection, but this was the first time where such a clear link has been made and will pave the way for future work to reduce sample errors and therefore LOS.

Regarding countermeasure 2, from the online survey (six responses), some agreed that the process was easier, while some reported it was more difficult for female patients; however, this was not deemed significant enough to revert to the previous process. The old process ease of use was scored 9.2, whereas the new process was scored 8.2. In terms of the patient audit of urine sample collection, it is difficult to compare two one-off audits as they do not account for normal variation; therefore, we cannot say that this countermeasure made an impact. PDSA 4 has been handed over to the infection control team and a doctor in training who continue to work on this.

Finally, countermeasure 3 saw Urology SDEC attendances increase from 17 to 32 per month over 4 months between November and February.

In terms of balancing measures, errors decreased from 2.3% (July to December 2024) to 1.8% (January 2024), demonstrating that the interventions did not unintentionally increase sample errors. Staff satisfaction remained high at over 8/10 from the survey. Therefore, there was not an adverse effect based on the balancing measures agreed. There was no balancing measure for countermeasure 3.

### Sustainability plan

A robust sustainability plan has been established to ensure the long-term impact of this project. Key interventions have been embedded into standard practice, such as the new urine sample process, and the SDEC triage change has formed ED policy. The Infection Prevention and Control (IPC) nurses have taken over the responsibility of maintaining sustainment, ensuring dedicated oversight. Regular audits and ongoing data collection on sample errors, the number of samples sent for eligible patients and the number of patients triaged to urology SDEC will enable continuous monitoring and improvement. Additionally, QI training has been delivered to IPC nurses who are taking over the project.

## Lessons and limitations

The project team experienced various limitations and lessons learnt. The unavailability of the LOS patient list with discharge dates meant the team were unable to use weekly data for bed days. This reduced the project team’s ability to identify a positive step change in LOS as the monthly data were not responsive enough to the rapid improvements made on the project. In addition, countermeasure 2 carried out two audit cycles; therefore, a run chart was not available, making it impossible to identify whether a step change occurred.

Another limitation was the limited capacity of doctor in training to take forward actions, such as engaging of peers and gathering data. Often, they dedicate free time to improvement projects and do not have protected work time for healthcare improvement. However, the doctors on this project were able to support with audits, giving views in meetings and driving forward SDEC changes.

By allocating a dedicated improvement coach (also acted as project manager) to the project, it enabled sustained momentum and changes to be made more quickly during a period of operational pressures. However, the coach stepped away from the project after 12 weeks as planned, which meant future PDSA cycles were continued in subgroups and not as easy to track.

Sharing the project at clinical governance meetings encourages and inspires other clinicians to pick up future cycles of change after the project has moved into monitoring.

Having a wide variety of engaged stakeholders from a variety of professions brought in a range of ideas which ultimately determined the direction and success of the project.

## Conclusion

The project’s aim was ambitious with the outcome measure being reducing bed days for UTI patients aged 16–65. We cannot say whether it met the target as there are insufficient monthly data points to draw conclusions. However, the process measure of reducing sample errors due to white tops was achieved and is expected to have a positive impact on LOS. Further change cycles are being carried out to increase urine samples sent and the team is waiting for data on the new process of nurses triaging directly to SDEC.

Previously, there were no specific studies on LOS for UTI patients available and this project adds some new information. First, the audit regarding errors evidenced a clear link between urine sample errors and significant prolongation of LOS of UTI patients. This confirmed the urology team’s observations, led to a tangible improvement and will pave the way for more sample error reduction work. Second, interrogating the process and including the physical materials demonstrated a more lasting and impactful result than solely focusing on human factors and decision-making in relation to LOS. Finally, by using A3 Thinking methodology ^9^, the newly formed team were able to align themselves quickly to the vision, define the scope based on data and work methodically to maximise the value of the time they invested in this piece.

In terms of cost reduction, the project produced a cost efficiency improvement without direct budget reduction of approx. £30 000 yearly due to the estimated reduction in bed days associated with urine sample errors. The change of urine sample collection process created a small cash-releasing saving of approx. £2000 per year due to moving from one cup, bag and tube to just one bottle.

In terms of sustainability, it is believed that because the team introduced process changes, the project is likely to be sustainable. A doctor in training is revisiting one countermeasure, the project lead continues to work in ED and is monitoring results of the new triage pathway and the data are being monitored and escalated where appropriate. Potential risks to sustainability include personnel changes, changes in the organisation’s priorities and operational pressures.

## Data Availability

Data are available upon reasonable request.
